# Comprehensive Utilization of Industry By-Products in Precast Concrete: A Critical Review from the Perspective of Physicochemical Characteristics of Solid Waste and Steam Curing Conditions

**DOI:** 10.3390/ma17194702

**Published:** 2024-09-25

**Authors:** Yang Shao, Zengqi Zhang, Xiaoming Liu, Lilei Zhu, Chun Han, Siyi Li, Weijie Du

**Affiliations:** 1 School of Metallurgical and Ecological Engineering, University of Science and Technology Beijing, Beijing 100083, China; 13635663944@163.com (Y.S.); 18241775538@163.com (S.L.); ustbu202140406@163.com (W.D.); 2 China MCC22 Group Corporation Ltd., No. 16 Xingfu Road, Fengrun District, Tangshan 064000, China; mcczhulilei@126.com (L.Z.); sr1021139304@163.com (C.H.)

**Keywords:** precast concrete, solid waste, hydration reaction, steam curing

## Abstract

Solid wastes have been widely used as a cement substitute in precast concrete. On the one hand, solid waste can effectively ameliorate a series of problems caused by steam curing. On the other hand, the use of solid waste can reduce the amount of cement used in the construction industry and reduce carbon emissions. However, due to the complexity of the steam curing system, the performance of precast concrete prepared under different steam curing conditions varies greatly. Moreover, there are a wide variety of solid wastes, and the differences in the physicochemical properties of different solid wastes are significant. Therefore, it is necessary to systematically determine the mechanism of action of commonly used solid wastes. In this paper, the steam curing system is introduced in detail, and the mechanism of action of solid waste in precast concrete is systematically summarized. It was found that an appropriate increase in the temperature and duration of steam curing facilitates the strength development of precast concrete. In addition, there is a difference in the effect of the addition of solid wastes on the early and late strength of precast concrete, which usually leads to a decrease in the demolding strength of precast concrete, but increases the late strength of precast concrete. This study provides a reference for rationally regulating steam curing systems and realizing the comprehensive utilization of solid wastes in precast concrete.

## 1. Introduction

Unlike traditional construction sites with complex environments, assembly buildings have the advantages of convenient construction and high productivity and therefore have been vigorously promoted and supported by China [1,2]. According to a document issued by China in 2016, it is necessary to vigorously develop assembly buildings and strive to make the floor area of assembly buildings reach more than 30% of the new building area by 2026. Precast concrete is the most basic unit of assembly buildings and is widely used in marine, road and other projects [3,4]. However, to avoid damage to the surface and corners of precast concrete during demolding, the demolding strength needs to reach 15 MPa or more than 80% of the design strength before the material can be demolded [5,6]. This requirement raises the threshold of precast concrete production.

To meet production needs, many scholars have attempted to use various methods to promote the rapid development of the early strength of precast concrete [7]. At present, the most popular methods are adding chemical admixtures and performing steam curing [8]. Chemical admixtures mainly include inorganic early-strengthening agents, organic early-strengthening agents and new nanonucleating early-strengthening agents [9,10]. Despite the fact that these chemical admixtures contribute to the early demolding strength of concrete, there are very obvious drawbacks [11]. Calcium sulfoaluminate in sulfoaluminate cement can effectively promote the rapid hydration of silica-alumina in silicate cement, but this effect is only obvious when the water–cement ratio is low [12,13,14]. This will increase the cost of production and add to the economic burden of enterprises [15]. Among the organic early-strengthening agents, triethanolamine has the best early-strengthening effect [16]. It can accelerate the cement hydration reaction by complexing the dissolved Al^3+^ and Fe^3+^ during cement hydration to promote the dissolution of C_3_A and C_4_AF [17]. However, triethanolamine has the disadvantage of requiring a small dosage, and it is difficult to determine the dosage required to promote coagulation. Among these nanomaterials, C-S-H seeds are considered to be among the most promising early-strengthening agents [18,19]. On the one hand, C-S-H seeds are able to accelerate the hydration of cement by providing nuclear supply sites for cement. On the other hand, the small particle size of C-S-H seeds can fill the pores in precast concrete [20]. However, C-S-H seeds have a limited effect on the demolding strength of precast concrete, and it is difficult to prepare precast concrete without steam curing by relying on C-S-H seeds alone [21]. In addition, due to the temperature sensitivity of chemical admixtures, the performance of precast concrete products varies greatly between summer and winter, which limits the use of chemical admixtures.

Steam curing is still the most common way to prepare precast concrete. Under high-temperature conditions, substances such as C_3_S and C_3_A in cement rapidly hydrate [22,23]. Precast concrete samples can reach a very high strength in a short time, thus meeting the demand for demolding [24,25,26]. However, high-temperature steam curing is prone to produce connection channels with large pore sizes, which can limit the application range and reduce the service life of precast components [27,28]. For this reason, many scholars are actively looking for ways to compensate for the lack of long-term performance and durability of precast components. Studies have shown that the use of solid wastes as supplementary cementitious materials is a feasible and effective way to replace cement [29,30]. On the one hand, solid waste has certain cementitious activity, which can be fully stimulated under the action of a high temperature and can compensate for the loss of strength caused by the reduction in cement content [31,32]. Therefore, solid waste is a substitute for excellent quality cement. On the other hand, it realizes the secondary use of solid waste and directly reduces environmental pollution. A reduction in the use of cement is accompanied by a reduction in carbon emissions [33].

In recent years, solid wastes have been continuously researched and developed and can be effectively applied in the production of precast components, but there are still many problems restricting the use of solid wastes. First, the steam curing system is complicated [34]. The hydration mechanism in precast concrete under high temperatures has not been fully clarified, and the difference in the performance of precast concrete under different steam curing conditions is obvious [35]. Second, there are many types of solid wastes, with obvious differences in their physicochemical properties and different ranges of application. Designing a more scientific steam curing system and utilizing solid wastes is highly important for the precast industry. For this reason, combined with the literature, this paper systematically reviews the effects of the steam curing system and the mechanism of solid wastes on the properties of precast concrete to provide a reference for the efficient utilization of solid wastes in precast concrete.

## 2. Steaming System

### 2.1. The Process of Steam Curing

**Figure 1:**
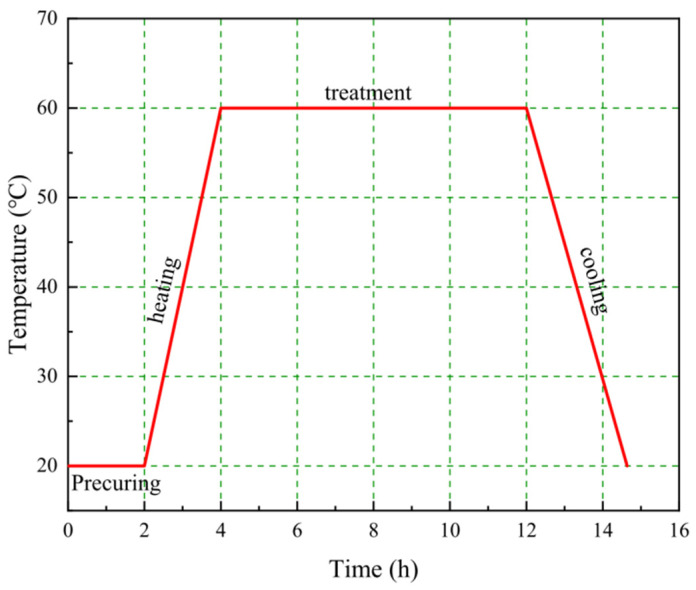
The process of steam curing [42].

**Figure 2:**
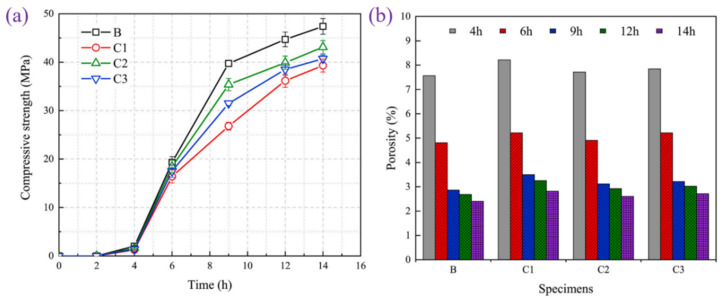
Variation in the mechanical and microscopic properties of precast concrete with time during steam curing: (**a**) Compressive strength [43]; (**b**) Porosity [44].

As shown in Figure 1, the entire steam curing system can usually be classified into four stages: the precuring stage, heating stage, treatment stage and cooling stage [36]. The precuring stage is designed to ensure that the precast concrete develops a certain level of strength, with the aim of mitigating the thermal damage caused to the precast concrete by steam curing. During the heating stage, the initial structure of the precast concrete begins to take shape gradually [37]. Due to the poor thermal conductivity of concrete, the temperature and water vapor conduction process take some time, so the internal temperature rise rate of precast concrete is relatively slow. The cement inside the precast concrete is not stimulated by high temperatures in time, and the rate of hydration is slow; at this time, the hydration product inside the precast concrete decreases, and the porosity increases. With the gradual increase in temperature, water vapor slowly enters the interior of the concrete [38]. Under the heating effect of high-temperature steam, the surface and interior of precast concrete begin to experience an obvious temperature gradient [39]. Figure 2 shows the variation in the compressive strength and porosity of precast concrete with time during the steam curing process. The cement is rapidly hydrated under the action of a high temperature, and a series of hydration products are rapidly generated, which leads to an improvement in the densification of the precast concrete and a rapid reduction in porosity while a certain degree of strength is formed. Shi et al. systematically investigated the changes in strength of precast concrete throughout a whole steam curing system [40,41]. During the treatment stage, the strength reached more than 85% of the demolding strength. Finally, during the cooling phase, the rate of generation of hydration products decreases as the temperature decreases, and water vapor diffuses outwards from within the concrete.

### 2.2. Steam Curing Parameters

#### 2.2.1. Precuring Time

The purpose of precuring is to increase the strength of precast concrete to resist thermal damage caused by steam curing. The precuring time is usually approximately 2–4 h. Prolonging the precuring time will result in the whole prefabrication process being elongated, which will affect the economic efficiency [45]. Similarly, shortening the precuring time can result in precast concrete that is susceptible to thermal damage during subsequent steam curing due to its low early strength [46].

#### 2.2.2. Temperature Rise/Cooling Rates

Studies on the temperature rise/cooling rates have focused on the effect on the pore structure. At the beginning, the strength of precast concrete is relatively low, and controlling the temperature rise/cooling rates essentially helps to ease the thermal damage caused by steam curing. If the temperature increase rate is too high, then the expansion of water and precast concrete caused by high temperatures may lead to an increase in precast concrete porosity. In severe cases, even cracks are produced. If the temperature cooling rate is too high, then the temperature difference between the inside and outside is large, which may likewise lead to the cracking of the precast concrete. The difference is that with the completion of steam curing, the precast concrete obtains a certain demolding strength, so the cooling rate has relatively little effect on the precast concrete. In addition, maintaining a certain temperature increase/cooling rate is also intended to speed up mold turnover and improve production efficiency. Therefore, the temperature increase/cooling rate is usually controlled in the range of 15–20 °C/h.

#### 2.2.3. Temperature during Steam Curing

**Figure 3:**
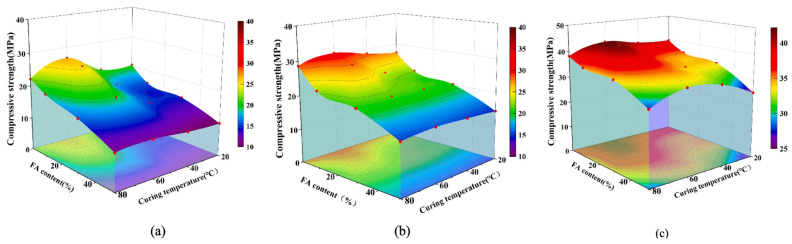
Variation in the compressive strength of FA-based precast concrete at different ages: (**a**) 36 h; (**b**) 3 d; (**c**) 28 d [2].

**Figure 4:**
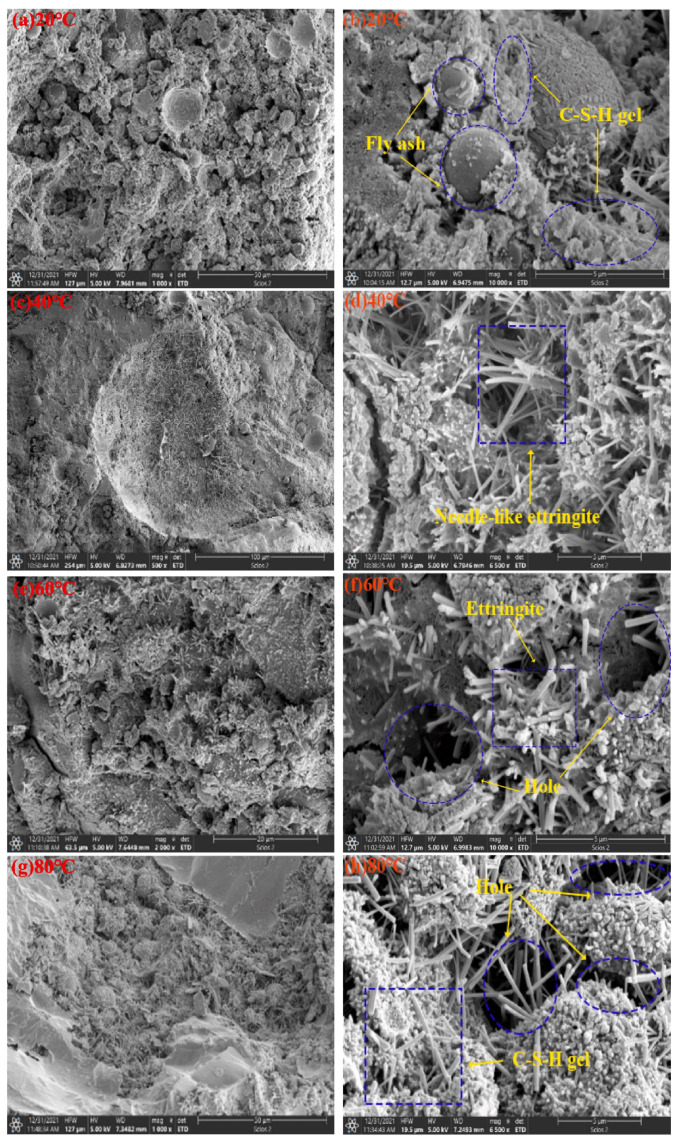
Microstructure of precast concrete with 30% FA incorporated at different temperatures: (**a**,**b**) 20 °C; (**c**,**d**) 40 °C; (**e**,**f**) 60 °C; (**g**,**h**) 80 °C [2].

At present, the steam curing temperature commonly used in experimental research is in the range of 45–90 °C. Figure 3 shows the strength changes in fly ash (FA)-based precast concrete from 36 h to 28 d at different steam curing temperatures. With an increasing temperature, the strength tended to first increase and then decrease [2]. Figure 4 shows the effect of the steam curing temperature on the microstructure of FA-based precast concrete. The precast concrete cured by steam at 40 °C was structurally denser and generated more hydration products than the standard cured (20 °C) concrete [2]. With increasing temperature, visibly large pores were observed in the 60 and 80 °C samples. This indicates that a high temperature accelerates the hydration of cementitious materials while causing irreversible damage to the microstructure of precast concrete. In addition, delayed ettringite formation occurs within 7 d after the steam curing of precast concrete above 60 °C, and the delayed generation of ettringite can be detrimental to the volumetric stability of precast concrete. In general, a temperature below 60 °C is more than a reasonable temperature for steam curing.

#### 2.2.4. Time of Steam Curing

**Figure 5:**
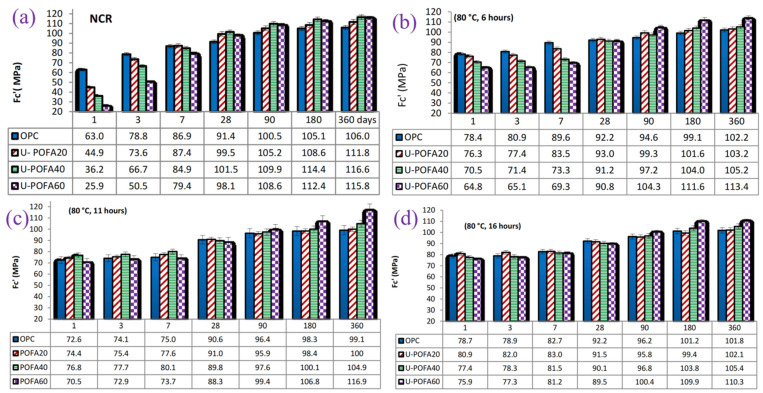
Compressive strength of POFA-based precast concrete under different conditions: (**a**) 27 °C, (**b**) 80 °C, 6 h; (**c**) 80 °C, 11 h; (**d**) 80 °C, 16 h [48].

**Figure 6:**
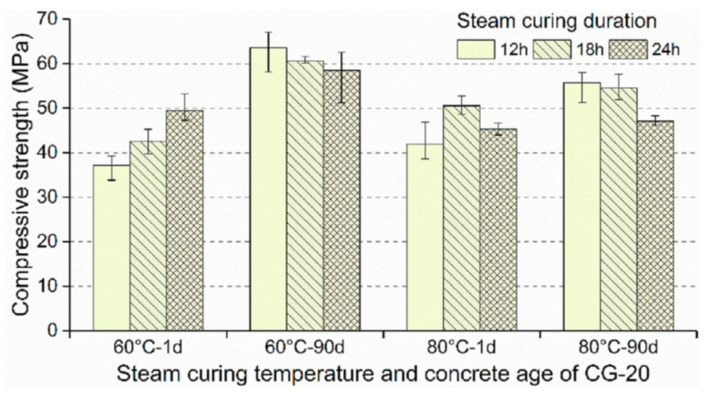
Compressive strength of 20% CG-based precast concrete under different steam curing times [49].

Figure 5 and Figure 6 show the compressive strengths of palm oil fuel ash (POFA) and coal gangue (CG)-based precast concrete at different steam curing times, respectively. Extending the steam curing time seriously affects the later strength of the precast concrete, but the effects on the early strength are different. Extending the curing time from 12 h to 24 h at 60 °C increased the early strength. However, at 80 °C, the 1 d compressive strength of POFA-based precast concrete decreases and then increases, and the 1 d strength of CG-based precast concrete increases and then decreases with increasing steam curing duration [47,48,49]. This means that the influence of curing time on the early strength is affected by a combination of factors, such as the steam curing temperature, the water–cement ratio, and the content and activity of the cementitious materials. Taken together, an appropriate extension of the steam curing time is conducive to improving the demolding strength. However, when the steam curing time exceeds a certain threshold, it may even be counterproductive. Therefore, the whole steam curing process is usually controlled within 24 h.

### 2.3. Problems in the Steam Curing System

Although high temperatures can promote the rapid development of early concrete strength to accelerate mold turnover and improve economic efficiency, there are also many disadvantages [50]. First, high temperatures consume a large amount of energy, which can cause severe environmental pollution. According to statistics, Steam curing of only 1 m^3^ of precast concrete consumes 30–40 kg of standard coal and emits about 50 kg of CO_2_ [51]. Second, although steam conservation accelerates the generation of hydration products at the initial stage, the migration of these hydration products to other places is relatively slow. Therefore, these hydration products are more likely to accumulate together to form coarse crystalline products, which hinder the subsequent hydration reaction [52,53]. Moreover, hydration products are more inclined to adhere to the surface of the cement particles rather than filling the pores of the precast concrete, which is unfavorable to the pore structure [54]. Finally, steam curing tends to form connected pore structures with large diameters, which is detrimental to volumetric stability [46].

## 3. Application of Solid Wastes in Precast Concrete

### 3.1. Deformation

Precast concrete first expands slightly during the precuring and heating stages. Precast concrete will experience significant shrinkage as the cement hydrates and water evaporates. The shrinkage of precast concrete must be controlled because the early strength is relatively low, and large deformations can lead to cracks [55]. Solid wastes can alleviate shrinkage by replacing cement, and the main mechanisms can be summarized as follows: (1) Solid wastes generally consume less water than cement. Using solid waste as a cement substitute will result in more water remaining inside the precast concrete, which can directly alleviate the drying problem of precast concrete [56]. (2) The pozzolanic reaction usually occurs after the cement hydration reaction, so there is a time lag between the two reactions, which prevents the precast concrete from shrinking drastically in a short time [57,58]. (3) The high content of SO_3_ in solid wastes such as FA can provide sufficient sulfur for the hydration reaction, which leads to an increase in the generation of ettringite. The expansion stress generated by ettringite generation can inhibit the drying effect [59]. (4) Solid wastes with low reactivity and small particle sizes can be used as inert filler materials to inhibit shrinkage [60].

**Figure 7:**
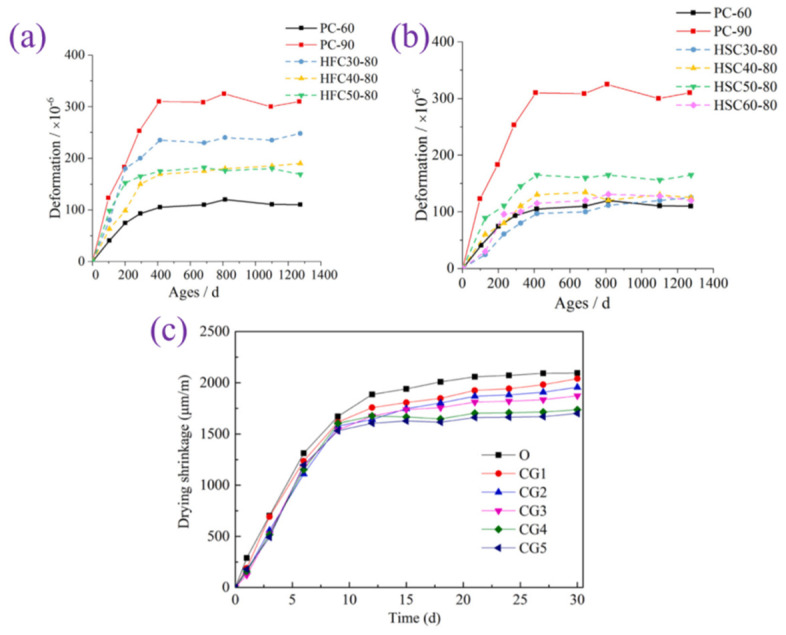
Effect of solid waste dosage on the degree of deformation: (**a**) FA; (**b**) GGBS [62]; (**c**) CG [56].

Figure 7 illustrates the effect of the admixture of solid wastes on the deformation of precast concrete. Zhuang et al. showed that when the admixture of FA or granulated blast furnace slag (GGBS) was less than 30%, the precast concrete underwent significant expansion [61,62]. However, when the dosage exceeded 40%, the results of continuing to increase the dosage of solid waste and changing the type of solid waste were minimal. Similar findings were reported by Yao et al. [56]. The degree of dry shrinkage of the precast concrete decreased continuously with the increasing CG replacement rate. The degree of dry shrinkage of the precast concrete decreased by 10.57%, 16.90%, and 18.66% when the CG content was 30%, 40%, and 50%, respectively. Obviously, when the amount of CG replacement is greater than 40%, the drying curves of the precast concrete highly overlap. This indicates that when the amount of ettringite in the CG is greater than 40%, continuing to increase the mixing amount has a low effect on the volumetric stability of the precast concrete.

### 3.2. Pore Structure

**Figure 8:**
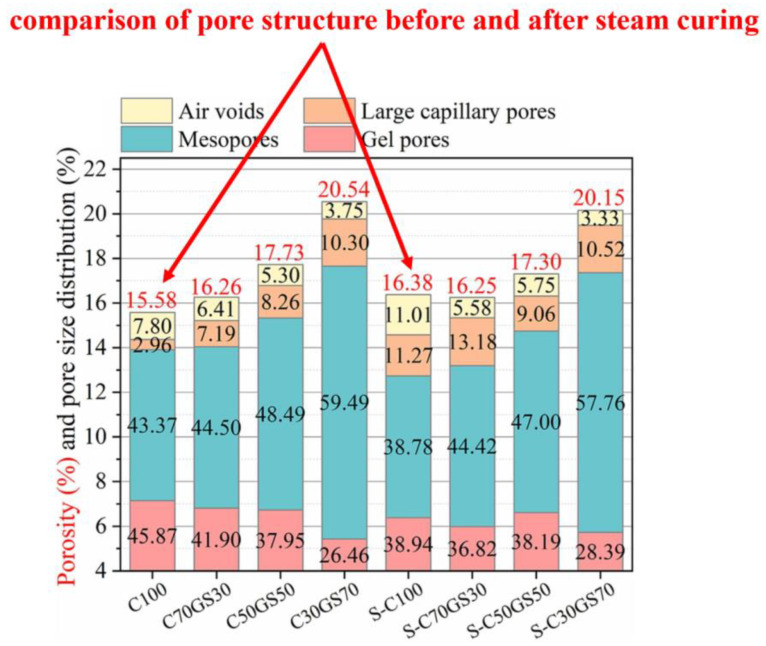
Differences in pore structure between standard cured concrete and precast concrete cured at high temperatures for 28 d [65] (C represents the amount of cement incorporated in the samples (%); GS represents the amount of slag incorporated in the samples (%); and S represents that the samples were steam cured).

In general, the pore size of concrete is often divided into four types: gel pores (0–20 nm), mesopores (20–50 nm), large capillary pores (50–200 nm) and air voids (>200 nm) [63,64]. Figure 8 shows that the proportion of gel pores and mesopores decreased after steam curing, while the percentage of large capillary pores and air voids increased [65]. The deterioration of the pore structure is one of the main reasons for the deterioration of precast concrete properties.

**Figure 9:**
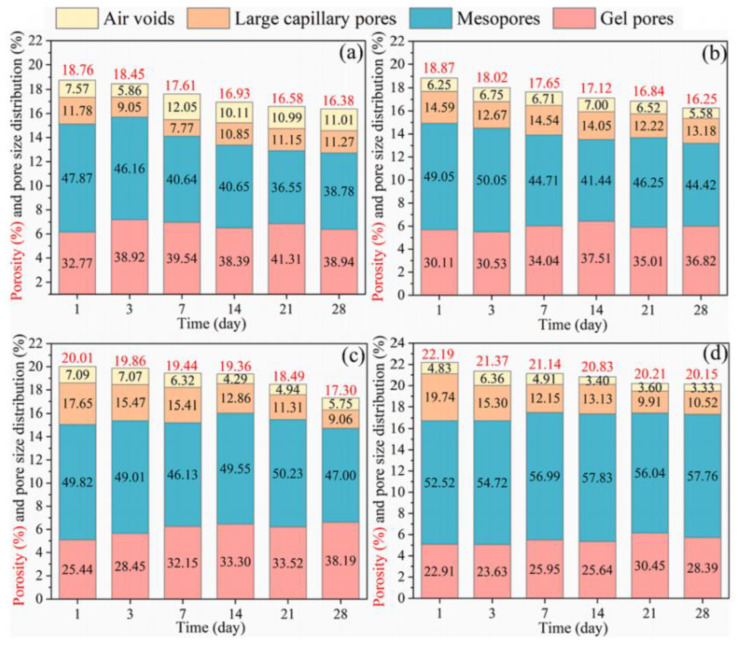
Effect of the GGBS dosage on the pore structure: (**a**) 0%, (**b**) 30%, (**c**) 50%, and (**d**) 70% [65].

As shown in Figure 9, the use of solid waste as a cement substitute can optimize the pore structure of precast concrete. Deng et al. examined the effect of the dose of GGBS on the pore structure from 1 to 28 d [65]. The total pore space of the precast concrete first decreased and then increased with the increasing GGBS admixture, but the percentage of large capillary pores and air voids in the precast concrete decreased continuously from 22.28% to 13.85%, which indicated that the precast concrete pore structure improved. On the one hand, solid wastes can fill the pores of precast concrete through the microaggregate effect, and on the other hand, C-(A)-S-H gels generated by the pozzolanic reaction of solid wastes with Ca(OH)_2_ can also refine the pore structure of precast concrete [66].

### 3.3. Cl^−^ Permeability

**Figure 10:**
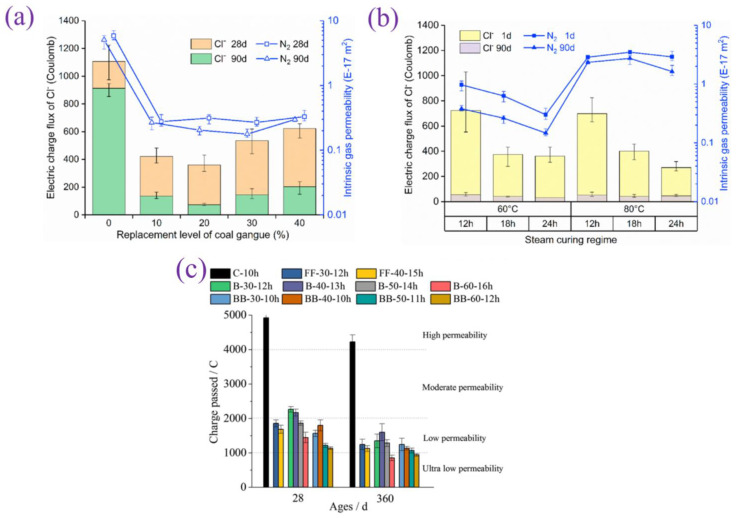
Effect of different solid wastes on the Cl^−^ permeability of precast concrete: (**a**,**b**) CG [49] and (**c**) FA and GGBS [43].

Three forms of Cl^−^ are broadly present in precast concrete: (1) Free Cl^−^: unhydrated Cl^−^ free in the pores of precast concrete; (2) Chemically bound Cl^−^: Friedel’s and Kuzel’s salts are representative chemical compounds; (3) Physically adsorbed Cl^−^: small amounts of Cl^−^ are physically adsorbed by raw materials or hydration products [67,68]. Therefore, to reduce Cl^−^ permeability, it is necessary to convert free Cl^−^ into chemical compounds or to adsorb it by physical means. Figure 10 shows the change in Cl^−^ permeability after the addition of different solid wastes, and it can be seen that the use of solid wastes can significantly reduce the Cl^−^ permeability. Solid wastes such as CG can physically adsorb Cl^−^ through the porous microstructure, which in turn leads to a decrease in the Cl^−^ permeability of precast concrete [49]. Moreover, the C-(A)-S-H gel hydration products also adsorb Cl^−^, and this adsorption effect is reversible. In addition, the SO_3_/Al_2_O_3_ ratio in cement substitutes is much lower than that in cement. This causes the substitution of SO_4_^2−^ by Cl^−^ in AFm-like, creating an environment conducive to the generation of Friedel’s and Kuzel’s salts, which will promote the conversion of free Cl^−^ [69]. Finally, there is a correlation between Cl^−^ permeability and pore structure. The decrease in Cl^−^ permeability is undoubtedly due to the improvement in the densification of the precast concrete pore structure after the incorporation of solid wastes [70].

### 3.4. Delayed Ettringite Formation

**Figure 11:**
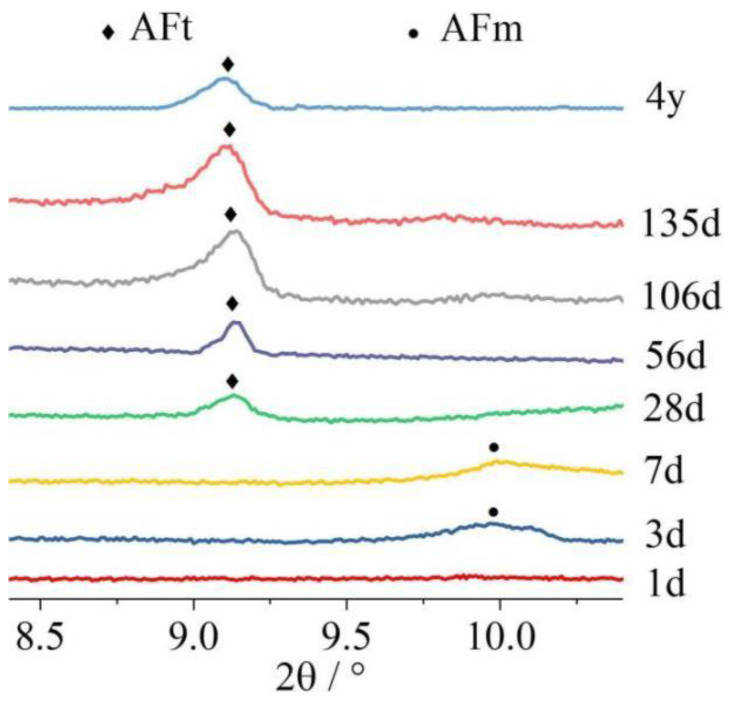
XRD patterns of samples prepared from pure cement at 90 °C [62].

**Figure 12:**
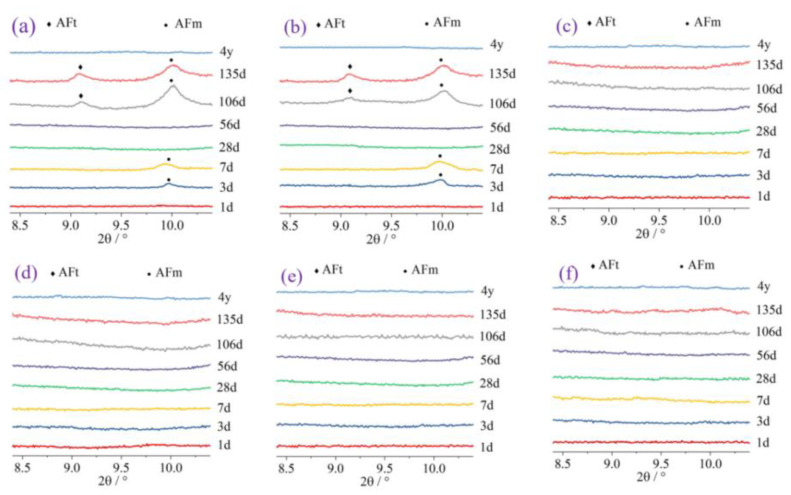
XRD patterns of FA- and GGBS-based precast concrete with different admixture doses at 80 °C: (**a**) 30% FA; (**b**) 50% FA; (**c**) 30% GGBS; (**d**) 40% GGBS; (**e**) 50% GGBS; (**f**) 60% GGBS [62].

Delayed ettringite formation is a harmful chemical reaction that occurs in precast concrete above 60 °C and with sufficient moisture [71,72]. First, under the action of a high temperature, ettringite decomposes into AFm, SO_4_^2−^ and Al^3+^, which are subsequently absorbed by C-S-H gels. After the end of steam curing, AFm, SO_4_^2−^, and Al^3+^ are released by the C-S-H gels, and ettringite is formed again [73]. However, C-S-H gels release ions at a slow rate, which leads to delayed ettringite formation [74]. Figure 11 shows the XRD pattern of the precast concrete of pure cement after 90 °C of steam curing. Typical delayed ettringite formation was observed, i.e., ettringite was produced after 7 d with a delay [62]. Delayed ettringite formation causes expansion damage to precast concrete and impairs the volumetric stability of precast concrete. Figure 12 shows the XRD pattern of precast concrete after the addition of more than 30% FA and GGBS at 80 °C. The delayed ettringite formation was delayed beyond 56 d in the samples with the addition of FA, whereas no delayed ettringite formation was found in the samples with the addition of GGBS for 4 years. Solid wastes can inhibit delayed ettringite formation by lowering the concentration of certain ions or increasing the binding difficulty of ions. First, the pozzolanic reaction of solid wastes consumes Ca(OH)_2_ to reduce Ca^2+^ and alkalinity in the system, which can inhibit the occurrence of delayed ettringite formation. Moreover, the microaggregate effect of solid wastes can reduce the connected porosity of precast concrete, which leads to difficulty in supplying free water to the precast concrete in a timely manner, and sufficient moisture is necessary for delayed ettringite formation. Furthermore, in terms of the elemental composition, the SO_3_/Al_2_O_3_ ratio of solid wastes is much lower than that of cement [75,76]. Therefore, the S content in the system decreases, and the Al content increases after the incorporation of solid waste.

### 3.5. Compressive Strength

**Figure 13:**
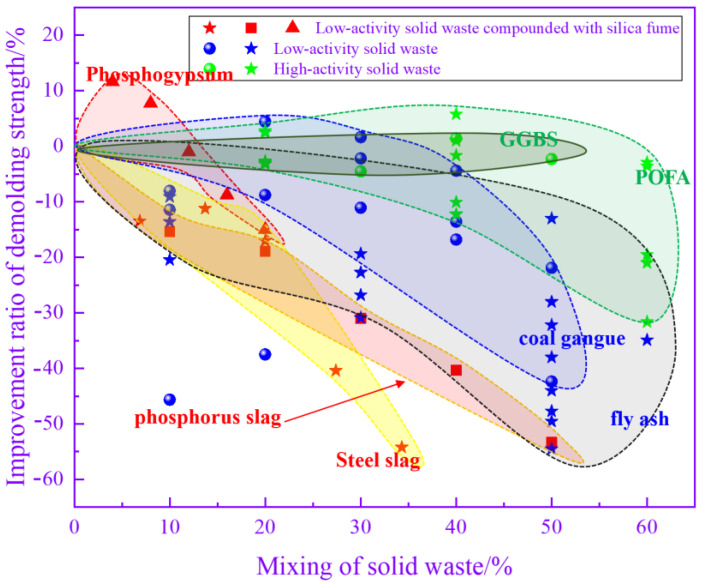
Relationship between the mixing of solid waste and the enhancement ratio of the demolding strength.

Demolding strength is a prominent criterion for measuring precast concrete. The higher the demolding strength is, the more difficult it is for precast concrete to be damaged during demolding and transportation. Figure 13 illustrates the relationship between the admixture of solid waste and the percentage of precast concrete demolding strength improvement, which can be classified into three categories according to the mode of solid waste incorporation and the level of pozzolanic activity. The first is the use of low-activity solid waste as a substitute for cement, which mainly includes FA, iron tailing powders and CG. The demolding strength decreases with increasing low-activity solid waste admixture. When the admixture of low-activity solid waste is 20%, the decrease in the demolding strength reaches 20%. Since the degree of reaction of low-activity solid waste is much lower than that of cement, it is difficult to rely on weak pozzolanic reactions to compensate for the decrease in strength caused by the replacement of solid waste with cement. The second type mainly involves mixing low-activity solid wastes such as phosphogypsum, phosphorus slag and steel slag with silica fume. This type of approach has a long steam curing period and a high temperature. As the admixture of phosphogypsum increased from 0 to 20%, the demolding strength of the precast concrete tended to increase and then decrease. The greatest increase in the demolding strength of the precast concrete was observed at a dosage of approximately 5%, which reached 10%. There is a synergistic effect between phosphogypsum and other high-activity cementitious materials. After compounding, the activity of both components is fully activated, which improves the demolding strength of precast concrete. Above 15%, the demolding strength started to decrease when the dose continued to increase. The effect of steel slag and phosphorus slag combined with silica fume is different from that of phosphogypsum. As the replacement ratio of steel and phosphorus slag increases, the demolding strength continues to decrease. When the dose is greater than 25%, the decrease in the demolding strength reaches more than 20%. As the proportion of low-activity solid waste increases, the total gelling activity in the system continues to decrease, which leads to a decrease in the demolding strength. The third category is the use of highly reactive solid wastes, mainly GGBS and POFA, to replace cement. When the dosage of highly active solid waste is lower than 40%, the loss of precast concrete demolding strength can be controlled within 10% by changing the steam curing system. This is because solid wastes such as GGBS have strong pozzolanic activity, and in the early stage of the response, they can rapidly undergo pozzolanic reactions to generate C-(A)-S-H gels to alleviate the strength reduction caused by the decrease in cement. Overall, the incorporation of solid wastes can adversely affect the demolding strength. In addition to adjusting the steam conservation conditions, increasing the fineness of solid waste is a common method of mitigating the adverse effects of solid waste on precast concrete. After doubling the specific surface area, the demolding strength of precast concrete prepared with an equal amount of GGBS under the same steam curing conditions increased by 20% [43].

**Figure 14:**
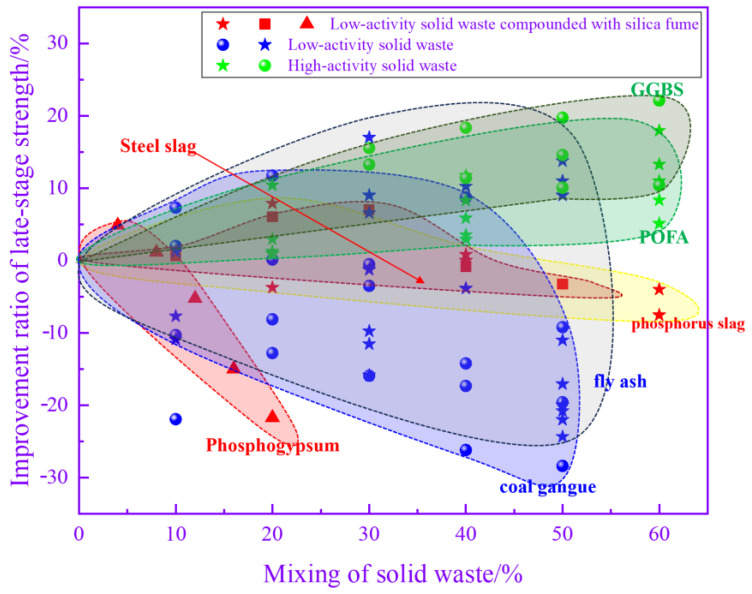
Relationship between the mixing of solid waste and the enhancement ratio of late-stage strength.

Figure 14 illustrates the relationship between the admixture of solid wastes and the percentage of the late strength enhancement of precast concrete. When low-activity solid wastes such as FA are added, the percentage of late strength enhancement fluctuates over a wide range. The later strength of precast concrete is determined by a number of factors, such as the steam curing conditions, the activity of the solid waste, and the admixture of the solid waste. The data fluctuate over a wide range due to differences in the steam conservation conditions of the data being analyzed. In the case of phosphogypsum compounded with silica fume, the improvement ratio of the late-stage strength increases and then decreases as the cement replacement specific gravity increases. A negative increase in strength was observed after the total dosage of phosphogypsum and silica fume exceeded 10%. After that, the late-stage strength of the precast concrete decreased rapidly with a further increase in the admixture. In contrast, the proportion of the late strength improvement of precast concrete prepared by compounding steel slag, phosphorus slag, and other solid wastes with silica fume is stabilized within certain limits. Even when the substitution rate reaches 50%, the decrease is still less than 10%. This may be because compared to phosphogypsum, the synergistic effect between silica fume and low-activity solid waste such as steel slag is more obvious at high temperatures. This leads to improvements in the late strength of precast concrete at high mixing ratios. For highly reactive solid wastes, the percentage of the late strength improvement of precast concrete increases and then decreases with the increasing admixture. At a dosage of approximately 40%, the percentage increase in the late strength is maximized, which is approximately 20%. The late strength of the precast concrete was still improved at a 60% admixture of highly reactive solid waste. The sustained pozzolanic reaction and microaggregate effect of highly reactive solid waste are fully utilized over an extended period. Thus, it has the effect of improving the late strength of precast concrete.

### 3.6. Common Solid Wastes

**Figure 15:**
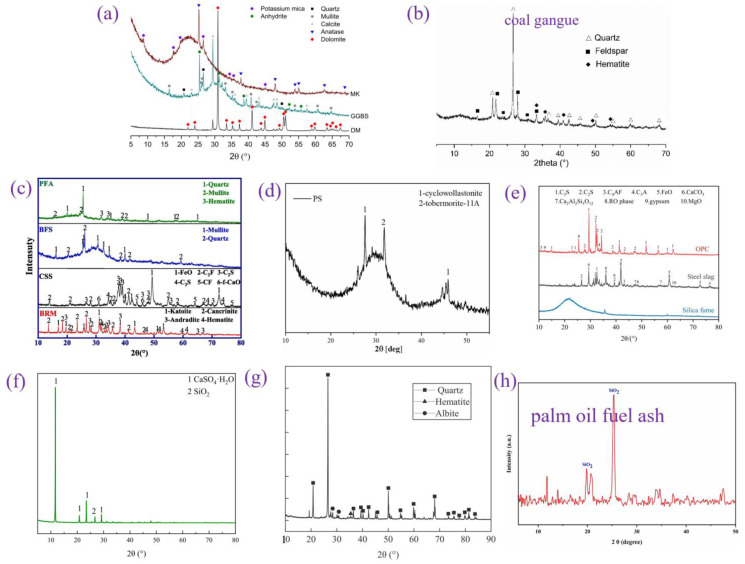
XRD patterns of common solid wastes: (**a**) GGBS [42]; (**b**) CG [49]; (**c**) FA and RM [79]; (**d**) phosphorus slag [80]; (**e**) steel slag [81]; (**f**) phosphogypsum [82]; (**g**) iron tailing powder [83]; and (**h**) POFA.

By reviewing the literature, a distinction was made between common solid wastes. Materials with calcium contents higher than 15% are called high-calcium solid wastes, and those with calcium contents lower than 15% are called low-calcium solid wastes. Figure 15 shows the XRD patterns of common solid wastes. Table 1 shows the mechanism of action and recommended dosage of solid wastes in the literature.

#### 3.6.1. High-Calcium Solid Wastes

High-calcium solid wastes include GGBS, red mud (RM), steel slag, phosphogypsum and phosphorus slag. The mineral composition of GGBS is significantly different from that of the other four solid wastes. On the one hand, the high percentage of amorphous objects in GGBS leads to a strong pozzolanic reaction, and on the other hand, the main phases of GGBS are calcium silicate and calcium silica-aluminate, which enables GGBS to exhibit excellent cementing activity in silicate- or alkali-inspired cements. In addition, the average particle size of GGBS is relatively small, and these characteristics make GGBS an excellent substitute for cement.

The low number of active components and the presence of large amounts of inert material in RM result in the low volcanic ash activity of RM. However, the unique composition of RM makes it highly alkaline. Numerous studies have shown that in alkaline environments, silica-alumina activity can be stimulated, which promotes the precipitation of C-S-H gels and the generation of ettringite in precast concrete. Thus, the high alkalinity of RM improves the demolding strength. Steel slag, phosphogypsum and phosphorus slag mainly rely on microaggregate effects and synergistic interactions with other cementitious materials to enhance the strength of precast concrete.

#### 3.6.2. Low-Calcium Solid Wastes

POFA is a low-calcium solid waste with high volcanic ash activity, and its unique physicochemical properties make it an excellent cement substitute [48,77]. On the one hand, a lower average particle size is conducive to maximizing the microaggregate effect. On the other hand, the high percentage of amorphous objects in POFA means that POFA contains a large amount of reactive silica-aluminum substances.

Iron tailing powder, FA and CG are low-calcium solid wastes with relatively low reactivity. These solid wastes improve the properties of precast concrete mainly through the microaggregate effect. Additionally, B.V. Vaasudevaa et al. reported that C-S-H gels are more prone to precipitate when the Ca^2+^ content is low [78]. This is beneficial for the development of precast concrete strength. The reaction of low-calcium solid wastes with Ca(OH)_2_ can produce C-S-H gels with lower Ca/Si ratios, which is one of the reasons why low-calcium solid wastes can improve strength.

**Table 1:** Mechanisms of action and incorporation of solid wastes reported in the literature.

Project	SO_3_/Al_2_O_3_ Ratio	Mechanism of Action	Recommendation Method of Incorporation	Recommendation Dosage/%	Reference
GGBS	0.131–0.309	Microaggregate effect, pozzolanic reaction, dilution effect.	Can be mixed separately.	30–40	[39,43,58]
POFA	0.021–0.058			30–40	[48,77,84]
CG	0.018–0.022	Microaggregate effect, dilution effect.		20–30	[49,56]
FA	0.011–0.059			20–30	[37,39,46]
Iron tailing powder	0.045–0.094			10–20	[57,85]
Phosphorus slag	0.283–0.780			10–15	[17,86,87]
Phosphogypsum	51.4–77.16	Microaggregate effect, dilution effect, synergistic effects with highly active supplementary cementitious materials such as silica fume.	Mixed with other supplementary cementitious materials.	5–10	[82,88]
Steel slag	0.080–0.197			10–15	[81,89]
RM	0.027–0.033	Red mud can stimulate the activity of silica-alumina in cement through its high alkalinity, which can promote the development of early strength in precast concrete.		10–20	[42,90,91]

### 3.7. Mechanisms of Solid Wastes as a Cement Substitute

(1) Microaggregate effect. The pozzolanic reaction products of solid wastes and unreacted solid waste will fill the pores of precast concrete. This can optimize the pore structure and improve the properties of precast concrete [92,93]. (2) Dilution effect. After solid waste replaces cement, the amount of cement in the system decreases. This corresponds to an increase in the W/B ratio of the reaction, which is conducive to promoting the hydration reaction of the cement. (3) Pozzolanic reaction. Solid wastes can undergo pozzolanic reactions with Ca(OH)_2_. Products of pozzolanic reactions, such as C-S-A-H gels, can increase the strength of precast concrete [59,75]. (4) The addition of solid wastes can inhibit the occurrence of delayed ettringite formation, which helps to protect the internal structure and maintain the volumetric stability of precast concrete [76,94]. (5) The synergistic use of alkali-sulfate-alumino-silicate solid wastes can mutually stimulate the activity of these solid wastes, thus improving the performance of precast concrete.

## 4. Synergy between Solid Wastes at High Temperatures

**Figure 16:**
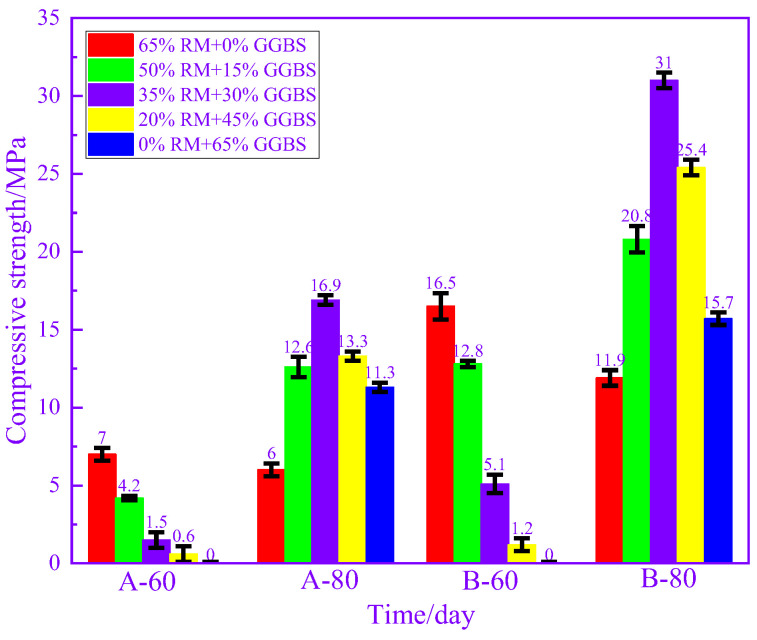
Demolding strength (14 h) of RM-GGBS-based precast concrete [42].

**Figure 17:**
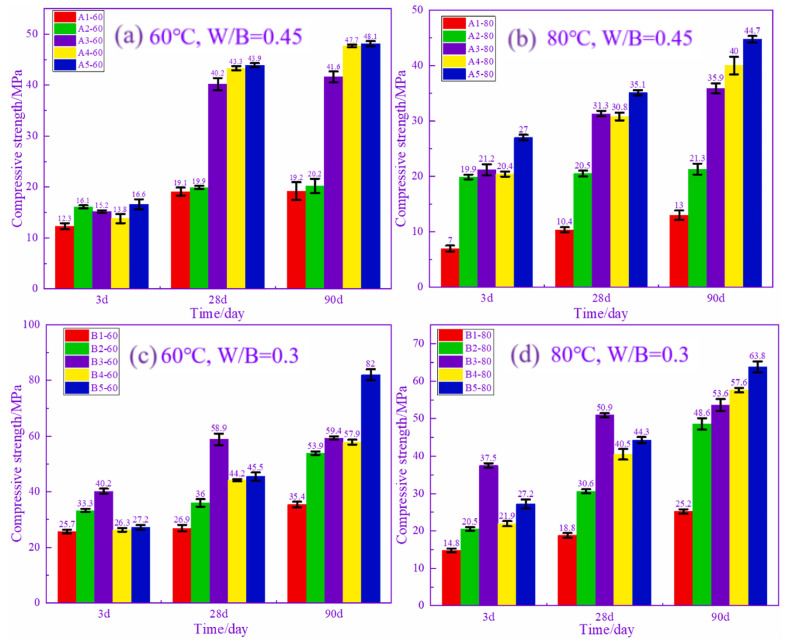
Compressive strength of RM-GGBS-based precast concrete within 90 d under different conditions [42].

At room temperature, synergistic effects between solid wastes have been widely reported and can be summarized and categorized into three types: alkali excitation, sulfate excitation and microaggregate effects [95,96,97]. However, synergistic effects between solid wastes at high temperatures have rarely been reported. Wang et al. examined the relationship between the mechanical properties of precast concrete and the cement-phosphogypsum-FA ternary cementitious composition [37]. After steam curing at 60 °C for 8 h, the precast concrete samples prepared with 8% phosphogypsum and 32% FA combined to replace cement had the best 28 d compressive strength, which was 10% higher than that of the cement samples. Han et al. found that the use of 50% iron tailing powder as a replacement not only led to a decrease in the compressive strength and Cl^−^ permeation resistance of precast concrete but also caused the deterioration of the pore structure. However, when the total substitution rate remains unchanged at 50%, the combination of iron tailing powder and GGBS can increase the demolding strength and the 90 d strength of precast concrete by 30% and can markedly improve the resistance to Cl^−^ penetration of precast concrete [57]. Most of the studies on the use of solid waste as a substitute for cement in precast concrete are limited to the amount of a particular type of solid waste incorporated and the steam curing temperature [58,98]. However, Shao et al. found that the synergistic effect between solid wastes at high temperatures was also affected by the steam curing temperature and the water/binder (W/B) ratio through a previous study [42]. Figure 16 demonstrates the demolding strength of RM-GGBS-based precast concrete. It can be seen that as the RM decreases and the amount of GGBS increases, the demolding strength of the precast concrete increases and then decreases at a high temperature (80 °C), and there is an obvious synergistic effect between RM and GGBS. At 60 °C, the situation was different with the change in admixture mix. The strength of precast concrete keeps decreasing, which means that the synergistic effect between RM and GGBS is mainly determined by temperature at this point. Figure 17 illustrates the 3–90 d compressive strength of RM-GGBS-based precast concrete. It can be seen that under the condition of a high W/B ratio, with the decrease in the RM admixture and the increase in the GGBS admixture, there is no obvious synergistic effect throughout the age of the precast concrete. However, when the W/B ratio is 0.3, with the change in admixture, the synergistic effect of the precast concrete under the two kinds of steam curing temperatures in the period of 3–28 d is obvious. This indicates that the synergistic effect between RM and GGBS at this time is mainly controlled by the W/B ratio. The synergistic effect between multiple solid wastes remains under high temperature steam curing conditions. And, it is consistent with the room temperature situation, which is affected by a combination of factors. In order to rationally utilize solid wastes and reduce environmental pollution, it is necessary to thoroughly study the synergistic effect of multiple solid wastes under high-temperature steam curing conditions in terms of both the mechanism and the regulation method.

## 5. Conclusions

This paper systematically introduces a steam curing system and summarizes the mechanism of action of different solid wastes as cement substitutes to improve the mechanical properties and durability of precast concrete. This study provides a reference for realizing the efficient utilization of solid wastes in precast concrete, and the main conclusions are as follows:

(1) Early temperature and humidity field changes determine the evolution of precast concrete properties. Although high temperatures can accelerate the early hydration rate of cementitious materials, they also jeopardize the late strength and durability of precast concrete. Therefore, a reasonable steam curing system needs to be selected according to the actual situation.
(2) Solid waste as a cement substitute can improve the properties of precast concrete by maintaining volumetric stability, optimizing pore structure, increasing late strength and reducing Cl^−^ permeability. In addition, it can also reduce carbon emissions, save resources and protect the environment.
(3) The use of solid waste leads to a reduction in the demolding strength. When 30% of the cement was replaced with low-activity solid waste, the demolding strength decreased by 20%. When the substitution rate is less than 15%, the synergistic mechanism between the high-activity gelling material and the low-activity solid waste can increase the demolding strength, whereas a decrease in the total cementitious content is detrimental to demolding when the substitution rate is above 25%. The intense pozzolanic reaction between Ca(OH)_2_ and high-activity solid waste can boost the development of early strength, so incorporating 40% high-activity solid waste will only slightly reduce the strength.
(4) Solid waste can improve the late strength of precast concrete. Low-activity solid wastes contain little active cementitious material and are subject to a combination of steam curing conditions, water–cement ratios and admixtures when used as a cement substitute. At a total substitution rate of approximately 20% for low-activity solid wastes and high-activity cementitious materials, the late strength increased by 10%. At a substitution rate of less than 40%, a synergistic effect between the cementitious materials was still present, which contributed to the development of the late strength. When the high-activity solid waste dose is less than 60%, the sustained pozzolanic reaction results in a significant increase in the late strength.
(5) The synergistic effect between multiple solid wastes under room temperature conditions has been a hot research issue in the field of building materials. After the preliminary research, it was found that the synergistic effect between alkaline-sulfate-aluminosilicate solid waste remains under high temperature steam curing conditions. In order to reduce environmental pollution and to dissipate stockpiled solid waste, it is necessary to thoroughly study the synergistic mechanism of multiple solid wastes under high-temperature steam curing conditions as well as the regulatory mechanism.
(6) Synergistic effects between solid wastes are the key to maximizing the utilization of solid wastes under steam curing conditions, and synergistic effects between high doses of solid wastes should be controlled according to the chemical composition of the solid wastes.

## Data Availability

The original contributions presented in the study are included in the article, further inquiries can be directed to the corresponding authors.
